# HIF1/2α mediates hypoxia-induced LDHA expression in human pancreatic cancer cells

**DOI:** 10.18632/oncotarget.15266

**Published:** 2017-02-10

**Authors:** Xin-gang Cui, Zhi-tao Han, Shao-hui He, Xing-da Wu, Tian-rui Chen, Cheng-hao Shao, Dan-lei Chen, Ning Su, Yuan-ming Chen, Ting Wang, Jing Wang, Dian-Wen Song, Wang-jun Yan, Xing-Hai Yang, Tielong Liu, Hai-feng Wei, Jianru Xiao

**Affiliations:** ^1^ Department of Bone Tumor Surgery, Changzheng Hospital, Second Military Medical University, Shanghai, China; ^2^ Department of Urinary Surgery of Third Affiliated Hospital, Second Military Medical University, Shanghai, China; ^3^ Department of Pancreatic Surgery, the First Hospital of China Medical University, Shenyang, China; ^4^ Department of Pancreatic Surgery, Changzheng Hospital, the Second Military Medical University, Shanghai, China; ^5^ Department of Colorectal Surgery, Changzheng Hospital, Second Military Medical University, Shanghai, China; ^6^ Department of Spine Surgery, Ruikang Hospital, Guangxi University Of Chinese Medicine, Guangxi, China

**Keywords:** HIF-1, HIF-2, hypoxia, LDHA, pancreatic cancer

## Abstract

Glycolysis is a typical conduit for energy metabolism in pancreatic cancer (PC) due to the hypoxic microenviroment. Lactate dehydrogenase A (LDHA) catalyzes the conversion of pyruvate to lactate and is considered to be a key checkpoint of anaerobic glycolysis. The aim of the present study was to explore the mechanism of interactions between hypoxia, HIF-1/2α and LDHA, and the function of LDHA on PC cells by analyzing 244 PC and paratumor specimens. It was found that LDHA was over-expressed and related to tumor stages. The result of in vitro study demonstrated that hypoxia induced LDHA expression. To explore the relationship between HIF and LDHA, chromatin immunoprecipitation assay and luciferase assay were performed. The result showed that HIF-1/2α bound to LDHA at 89bp under the hypoxic condition. Furthermore, knockdown of endogenous HIF-1α and HIF-2α decreased the LDHA expression even in the hypoxic condition, which was accompanied with a significant decrease in lactate production and glucose utilization (p < 0.01). Immunofluorescence in the 244 specimens showed that HIF-1/2α was over-expressed and associated with LDHA over-expression (p < 0.0001). Forced expression of LDHA promoted the growth and migration of PC cells, while knocking down the expression of LDHA inhibited the cell growth and migration markedly. In summary, the present study proved that HIF1/2α could activate LDHA expression in human PC cells, and high expression of LDHA promoted the growth and migration of PC cells.

## INTRODUCTION

Pancreatic cancer (PC) is one of the most common malignant tumors in the digestive system, with high incidence and mortality worldwide [1]. It was reported that 48,960 new cases of PC patients were diagnosed in the United States in 2015, of which 40,560 patients died [2]. Despite improvements in early diagnosis, surgical technology and systemic chemotherapy for PC in recent years, the overall 5-year survival rate remains below 5% [3]. This disappointing survival rate even after margin-negative pancreatectomy indicates the high grade of malignancy of this disease. It is therefore necessary and urgent to gain a better understanding about the molecular mechanisms underlying PC initiation and progression so as to find more effective therapeutic strategies for PC [4, 5].

Hypoxia can be sensed by individual cells so that they undergo metabolic adaptations to compensate for inadequate O2 supply. A major intracellular adaptation to severe hypoxia is the transition from oxidative phosphorylation to glycolysis as the principal means of generating adenosine triphosphate (ATP) [6, 7]. Some studies have demonstrated that the development and progression of tumors depend on glycolysis even under normal oxygen concentrations, which is defined as the “Warburg effect” [8]. In the final step of glycolysis, lactate dehydrogenase A (LDHA) converts pyruvate to lactate. The expression of LDHA was found to be increased in various types of human cancers [9, 10], including PC [11]. Previous studies [11, 12] found that increased LDHA expression could promote PC cell proliferation, migration and invasion. In addition, the expression level of LDHA was closely associated with tumor size, TNM stage and prognosis in PC patients [12], indicating that LDHA may be a potential prognostic marker and therapeutic target of PC.

Hypoxia is an important characteristic of tumor microenvironments [13]. Hypoxia inducible factor (HIF) plays a key role in the adaptation of tumor cells to hypoxia, and is the most critical transcription factor mediating cell response to hypoxia. Previous studies have demonstrated that HIF-1 activity is the determining factor for tumor development, and is related to cancer invasion, metastasis and prognosis [14, 15]. By binding to hypoxia response elements, HIF activates the expression of genes encoding glycolytic enzymes aldolase A (ALDA), LDHA, phosphoglycerate kinase 1 (PGK1), and pyruvate kinase M [16]. The expression level of LDHA is always in line with HIF-1 expression. However, the molecular mechanisms underlying the interaction between HIF-1 and LDHA remains unclear, also there is no study to explore the relation between HIF-2 and LDHA and the underline mechanisms.

The aim of the present study was to gain a more comprehensive understanding about the relationship between hypoxia, HIF-1/2 and LDHA in PC by detecting the expression of HIF-1/2 and LDHA in a series of PC specimens. In addition, the specific binding site of HIF-1 and HIF-2 in the LDHA promoter region was identified, hoping that the result could provide a theoretical basis for designing novel therapeutic strategies for PC.

## RESULTS

### LDHA is associated with the progression of PC

**Figure 1:**
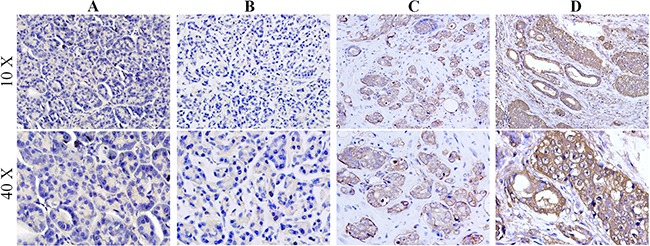
LDHA is associated with the progression of pancreatic cancers Immunohistochemical staining for LDHA was performed in pancreatic cancer. **A**. The para-tumor tissue exhibited low LDHA staining negative (0), **B**. weak (1+), **C**. moderate (2+) and **D**. strong (3+)

Knowing that the expression level of LDHA is increased in human PC and related to tumor stages [11], we detected the expression of LDHA in the 244 primary PC and paired para-tumor normal tissues by IHC staining (Figure 1). The results showed that LDHA was mainly expressed in the cytoplasm of tumor cells and significantly up-regulated in PC tissues as compared with the controls. Furthermore, the statistical results showed that the expression level of LDHA was positively correlated with disease stage and size, and increased LDHA expression was also correlated with decreased tumor differentiation (Table 1).

**Table 1:** Correlations between LDHA expression and clinicopathologic features in patients with pancreatic cancer

Variable	n	LDHA			P
		1+	2+	3+	
Pathological grading					
I	53	50	3	0	<0.001
II	145	67	72	6	
III	46	7	20	19	
Invasion depth					
T2	54	27	19	8	0.003
T3	169	93	65	11	
T4	21	4	11	6	
Lymph node metastasis					
N0	115	57	44	14	0.643
N1	129	67	51	11	
Distant metastasis					
M0	235	121	90	24	0.540
M1	9	3	5	1	
Tumor Size					0.102
≤6cm	57	36	17	4	
>6cm	187	88	78	21	
Gender					
M	141	70	56	15	0.908
F	103	54	39	10	
Age(years)	244	52.8±12.7	54.1±11.7	51.6±12.6	0.574

*means *p* < 0.05

### Hypoxia induces LDHA over-expression in human PC cells

**Figure 2:**
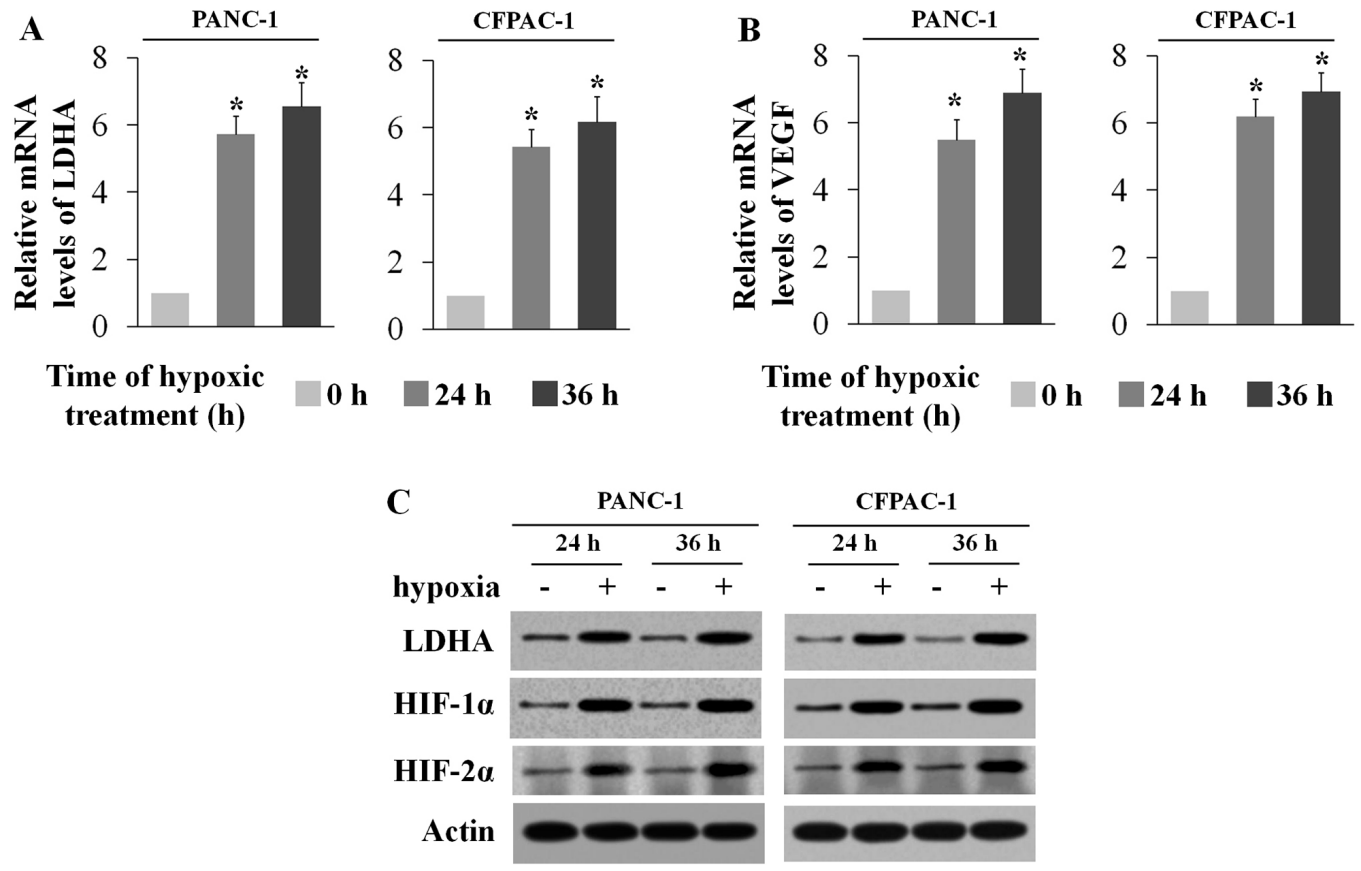
Hypoxia induces LDHA expression in human pancreatic cancer cell lines Human pancreatic cancer cell lines PANC-1 and CFPAC-1 cells were cultured under the hypoxic condition for the indicated time periods. **A**. The mRNA expression levels of LDHA in these cells were determined by RT-PCR. **B**. The mRNA expression levels of VEGF in these cells were determined as a positive control. **C**. The HIF-1α, HIF-2α and LDHA protein levels in these cells were determined by Western-blot assays. Data are presented as mean ± SD (n = 3). *: p < 0.01, Student's t-test.

Hypoxia is a hallmark of PC and other solid tumors. Interestingly, we found that LDHA expression was induced by hypoxia in PC. Human PC cell lines PANC-1 and CFPAC-1 were subjected to either hypoxia treatment (0.1% O2) or normoxia treatment (20% O2). Knowing that vascular endothelial growth factor (VEGF) is a hypoxia-inducible gene [17], we interacted HIF with HRE in the VEGF promoter and induced VEGF expression under hypoxia. The effect of hypoxia was confirmed by real-time PCR (Figure 2B) and Western-blot assays (Figure 2C). It was found that LDHA mRNA levels were significantly increased (p < 0.01) in both PANC-1 and CFPAC-1cells cultured under the hypoxic condition (Figure 2A). This hypoxia-induced LDHA expression was further confirmed by Western-blot assays (Figure 2C).

### HIF-1α and HIF-2α bind to HRE-D in the LDHA promoter under the hypoxic condition

**Figure 3:**
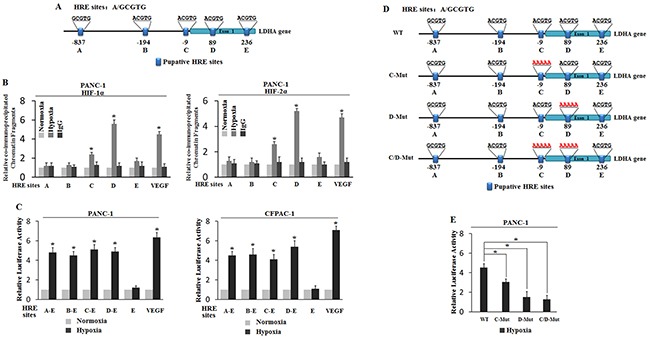
Hypoxia transactivates hypoxia-responsive elements (HREs) in the LDHA promoter through HIF-1α and HIF-2α **A**. The human LDHA gene contains 5 putative HREs in its promoter region. **B**. Both HIF-1α and HIF-2α bind to the HRE-D site under the hypoxic condition in PANC-1 cells as determined by ChIP assays. Cells were cultured under the hypoxic or normoxic condition for 36 h before assays. The HRE site in the VEGF promoter serves as a positive control. The amount of DNA fragments pulled down was determined by RT-PCR. **C**. Hypoxia activated the luciferase activity of reporter vectors containing HRE-D sites in the LDHA promoter. PANC-1 and CFPAC-1 cells were transfected with the luciferase reporter vectors, and then subjected to hypoxic or normoxic treatment for 36 h before measuring luciferase activities. Luciferase reporter vector containing the HRE site in the VEGF promoter was included as a positive control. **D**. Mutant HER C and HER D to 5′-AAAAA-3′. **E**. Luciferase activity after HER C and HER D mutation. Data are presented as mean ± SD (n = 3). *: p < 0.01 (Student's t-test).

HIFs are heterodimeric transcription factors composed of α-subunit and β-subunit of helix-loop-helix-PAS family proteins. HIFs bind to DNA containing a hypoxia-responsive element (HRE; 5 -G/ACGTG-3) dependent on the subunit HIF-1α and HIF-2α [18]. To investigate whether transcriptional induction of LDHA by hypoxia was mediated by HIFs, we searched for the HRE consensus sequence in the promoter region of the LDHA gene from 1863bq upstream of the transcriptional site to exon 1. Five putative HRE sites (HRE A, HRE B, HRE C HRE D and HRE E) were identified in the promoter region (Figure 3A). To investigate whether the hypoxia-induced LDHA expression was mediated by HIF-1α or HIF-2α, chromatin immunoprecipitation (ChIP) assay was employed to determine whether HIF-1α and HIF-2α physically could bind to HRE in the LDHA promoter. PANC-1 cells were cultured under the normoxic or hypoxic condition for 36 h, and ChIP assay was performed with an antibody against HIF-1α or HIF-2α. The quantity of chromatin fragments was determined by quantitative real-time PCR. The chromatin fragments containing HRE-D were pulled down by the antibody against HIF-1α or HIF-2α in PANC-1 under the hypoxic condition but not normoxic condition (Figure 3B). Interestingly, no clear immunoprecipitation of the chromatin fragments containing HRE A, HRE B, HRE C and HRE E by the antibody against HIF-1α or HIF-2α was observed in PANC-1 under the hypoxic or normoxia condition (Figure 3B). These results demonstrated that both HIF-1α and HIF-2α interacted with HRE-D in the LDHA promoter under the hypoxic condition.

### LDHA is transcribed under the hypoxic condition due to HRE-D in PC cells

To investigate whether these putative HREs accounted for the hypoxia-mediated induction of LDHA, the DNA fragments containing HRE A-E, HRE B-E, HRE C-E, HRE D-E and HRE E were inserted into a pGL3 luciferase reporter plasmid, and the DNA fragment containing HRE in the VEGF promoter was inserted into a pGL3 luciferase reporter plasmid to serve as a positive control. PANC-1 and CFPAC-1 were transiently transfected with these plasmids respectively, and PRL-SV40-TK plasmids were co-transfected as an internal standard to normalize transfection efficiency. As shown in Figure 3C, the luciferase expression level of the reporter plasmids containing the HRE from the VEGF promoter and the HRE A-E, HRE B-E, HRE C-E and HRE D-E was enhanced in the hypoxia groups, but no significant effect on HRE E was observed. Also, there was no significant difference in luciferase change in the normoxia condition whether the VEGF promoter or LDHA promoter was transfected. Furthermore, we mutated HRE C and HRE D sequences and found that the luciferase activity was significant decreased when HRE D was mutated (Figure 3D). These results indicate that the hypoxia-induced LDHA expression depended on the promoter containing functional HRE-D.

### HIF-1α and HIF-2α regulates LDHA expression at a transcriptional level

**Figure 4:**
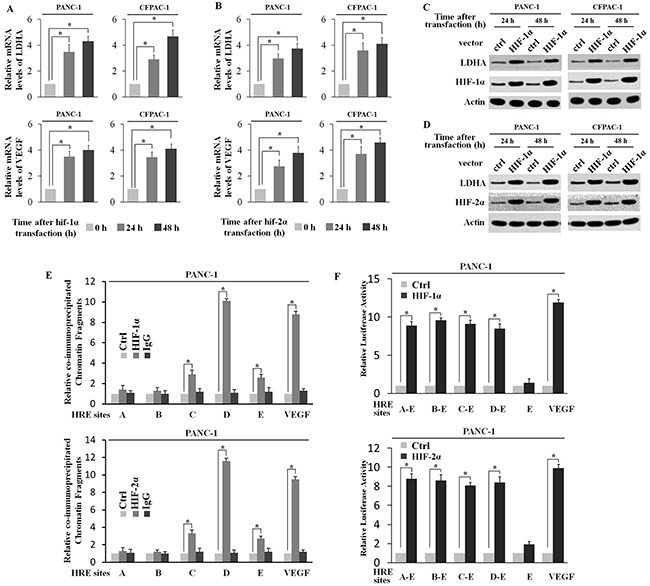
HIF-1α and HIF-2α transcriptionally regulate LDHA expression **A**. and **B**. Ectopic HIF-1α and HIF-2α expression increases LDHA and VEGF mRNA expression levels in PANC-1 and CFPAC-1 cells. The mRNA expression levels of LDHA and VEGF were determined by RT-PCR and normalized with actin 24 h or 48 h after HIF-1α or HIF-2α transfection. **C**. and **D**. Ectopic HIF-1α and HIF-2α expression increased LDHA protein levels in PANC-1 and CFPAC-1 cells as determined by Western-blot assays. **E**. HIF-1α and HIF-2α bound to the HRE-D site in the LDHA promoter in PANC-1 cells transfected with HIF-1α or HIF-2α expression plasmids as determined by ChIP assays. The amount of DNA fragments pulled-down was determined by real-time PCR. The HRE site in the VEGF promoter serves as a positive control. **F**. HIF-1α or HIF-2α activated luciferase activity of the reporter vector containing the HRE-D site in the LDHA promoter in PANC-1 cells transfected with HIF-1α or HIF-2α expression plasmids. The luciferase reporter vector containing the HRE site in the VEGF promoter was included as a positive control. Data are presented as mean± SD (n = 3). *: p < 0.01 (Student's t-test).

To investigate whether both HIF-1α and HIF-2α could active the LDHA expression level, PANC-1 and CFPAC-1 cells were transfected with the plasmids expressing HIF-1α (pcDNA3.1-HA-HIF2α) and HIF-2α (pcDNA3.1-HA-HIF1α). The ectopic expression of either HIF-1α or HIF-2α clearly induced VEGF mRNA expression (Figure 4A and 4B). Ectopic HIF-1α and HIF-2α significant increased LDHA expression at both mRNA and protein levels (Figure 4A and 4B). The ectopic HIF-1α and HIF-2α expression in cells was confirmed by Western-blot assays (Figure 4C and 4D). Furthermore, the degree of pull-down of chromatin fragments containing HREs in the LDHA promoter by HIF-1α and HIF-2α was determined by ChIP assays in PANC-1 and CFPAC-1 cells. As shown in Figure 4E, chromatin fragments containing HRE D was co-immunoprecipitated with the HIF-1α or HIF-2α. These observations clearly showed that HIF-1α and HIF-2α interacted with HRE-D in the LDHA promoter (Figure 4E). In addition, PANC-1 and CFPAC-1 cells were co-transfected with pcDNA3.1-HA-HIF1α or pcDNA3.1-HA-HIF2α and the pGL3 reporter plasmids containing HREs in the LDHA promoter. Notably, ectopic HIF-1α or HIF-2α expression obviously trans-activated the reporter plasmids containing HRE A-E, HRE B-E, HRE C-E and HRE D-E, but had a minimal effect on the reporter plasmids containing HRE-E (Figure 4F). Collectively, these results clearly showed that HIF-1α and HIF-2α interacted with HRE-D to induce LDHA expression.

### HIF-1α and HIF-2α mediate hypoxia-induced LDHA expression

**Figure 5:**
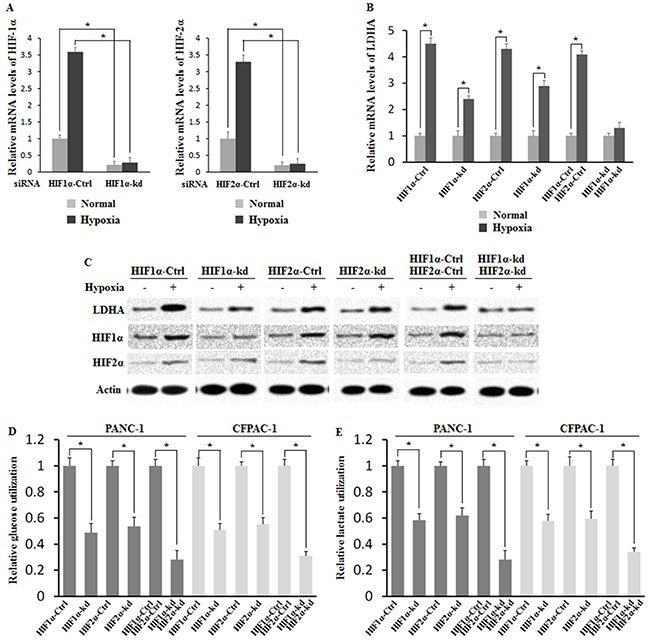
HIF-1α and HIF-2α mediate hypoxia-induced LDHA expression Knockdown of endogenous HIF-1α and HIF-2α largely reversed the hypoxia-induced LDHA expression in PANC-1 cells. Cells with knockdown of endogenous HIF-1α and HIF-2α by siRNA oligos or transfected with control siRNA were treated with hypoxia for 36 h. **A**. Knockdown of HIF-1α (left panel) and HIF-2α (right panel) in cells was confirmed at the mRNA level by RT-PCR and normalized with actin. **B**. The mRNA expression levels of LDHA were determined after knockdown of endogenous HIF-1α, HIF-2α or both HIF-1α and HIF-2α by RT-PCR and normalized with actin. **C**. LDHA level was determined after knockdown of endogenous HIF-1α, HIF-2α or both HIF-1α and HIF-2α by Western-blot assays and normalized with actin. **D**. The glucose utilization levels were determined after knockdown of endogenous HIF-1α, HIF-2α or both HIF-1α and HIF-2α. **E**. The lactate production levels were assayed after knockdown of endogenous HIF-1α, HIF-2α or both HIF-1α and HIF-2α. Data are presented as mean ± SD (n = 3). *: p < 0.01 (Student's t-test).

To investigate whether both HIF-1α and HIF-2α could mediate hypoxia-induced LDHA expression, endogenous HIF-1α or HIF-2α alone, or both were knocked down in PANC-1 cells by siRNA. Knockdown of endogenous HIF-1A and HIF-2α were determined by RT-PCR and Western-blot (Figure 5A and 5C). It was found that knockdown of endogenous HIF-1α or HIF-2α alone largely decreased the LDHA expression in the hypoxic condition, but no obvious effect was observed in the normoxia condition (Figure 5B and 5C). In addition, knockdown of HIF-1α decreased the LDHA expression more effectively as compared with knockdown of HIF-2α, but there was no significant difference between these two transcription factors. Furthermore, knockdown of both HIF-1α and HIF-2α in PANC-1 cells reversed the hypoxia-induced LDHA over-expression, and this effect was more pronounced than knockdown of HIF-1α or HIF-2α alone. These results clearly demonstrated that hypoxia induced the LDHA expression through HIF-1α and HIF-2α, and this effect could be reversed by targeting both HIF-1α and HIF-2α.

In addition, we investigated the roles of HIF-1α and HIF-2α in regulating aerobic glycolysis via LDHA by analyzing the glucose utilization and lactate production after HIF knockdown in PANC-1 and CFPAC-1. It was found that knockdown of HIF-1α or HIF-2α in PANC-1 and CFPAC-1 significantly decreased glucose utilization and lactate production induced by hypoxia (Figure 5D and 5E). Furthermore, knockdown of HIF-1α and HIF-2α in PANC-1 and CFPAC-1 almost reversed the effect of glucose utilization and lactate production induced by hypoxia (Figure 5D and 5E).

### HIF-1α and HIF-2α over-expression is associated with LDHA over-expression in human PC specimens

**Figure 6:**
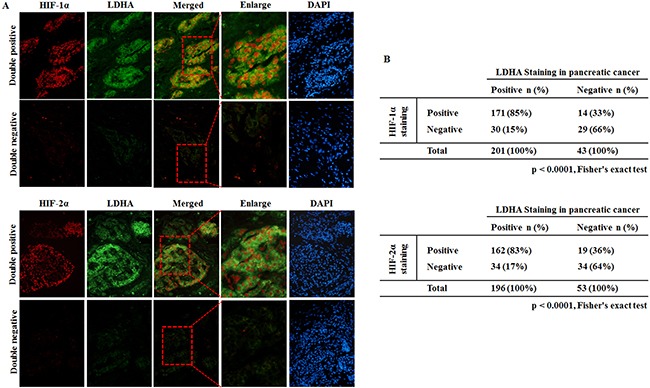
HIF-1α and HIF-2α over-expression is associated with LDHA over-expression in human pancreatic cancer specimens HIF-1α, HIF-2α and LDHA protein levels were determined by immunofluorescence staining in 244 cases of human pancreatic cancer specimens. **A**. Representative IFC staining results for HIF-1α and LDHA double positive (upper panels) or negative (lower panels) are shown. **B**. HIF-1α overexpression was associated with LDHA over-expression in human pancreatic cancer (p < 0.0001, Fisher exact test). **C**. Representative IFC staining results for HIF-2α and LDHA double positive (upper panels) or negative (lower panels) are shown. **D**. HIF-2α over-expression was associated with LDHA over-xpression in human pancreatic cancer (p < 0.0001, Fisher exact test).

To investigate whether HIF-1α and HIF-2α contributed to the increased expression of LDHA in human PC specimens, the expression levels of LDHA, HIF-1α and HIF-2α were determined in the 244 human PC specimens by double-labelling immunofluorescence. The representative immunofluorescence images of LDHA, HIF-1α and HIF-2α are shown in Figure 5. Consistent with our previous finding, a significant portion of PC specimens (201/244) showed positive LDHA staining. In addition, a significant portion of PC specimens showed positive HIF-1α staining (171/244) and positive HIF-2α staining (162/244) (Figure 6A). Fisher exact test showed that HIF-1α and HIF-2α over-expression was strongly associated with LDHA over-expression (Figure 6B) (p < 0.0001). HIF-1α and HIF-2α positive staining was observed in 85% or 83% cases with LDHA positive staining, but only in 14% or 19% cases with LDHA negative staining (Figure 6B). These results strongly suggest that the transcriptional induction of LDHA by hypoxia was due to both HIF-1α and HIF-2α. This may be an important mechanism accounting for the LDHA over-expression in human PC.

### LDHA promotes the growth and migration of PC cells

**Figure 7:**
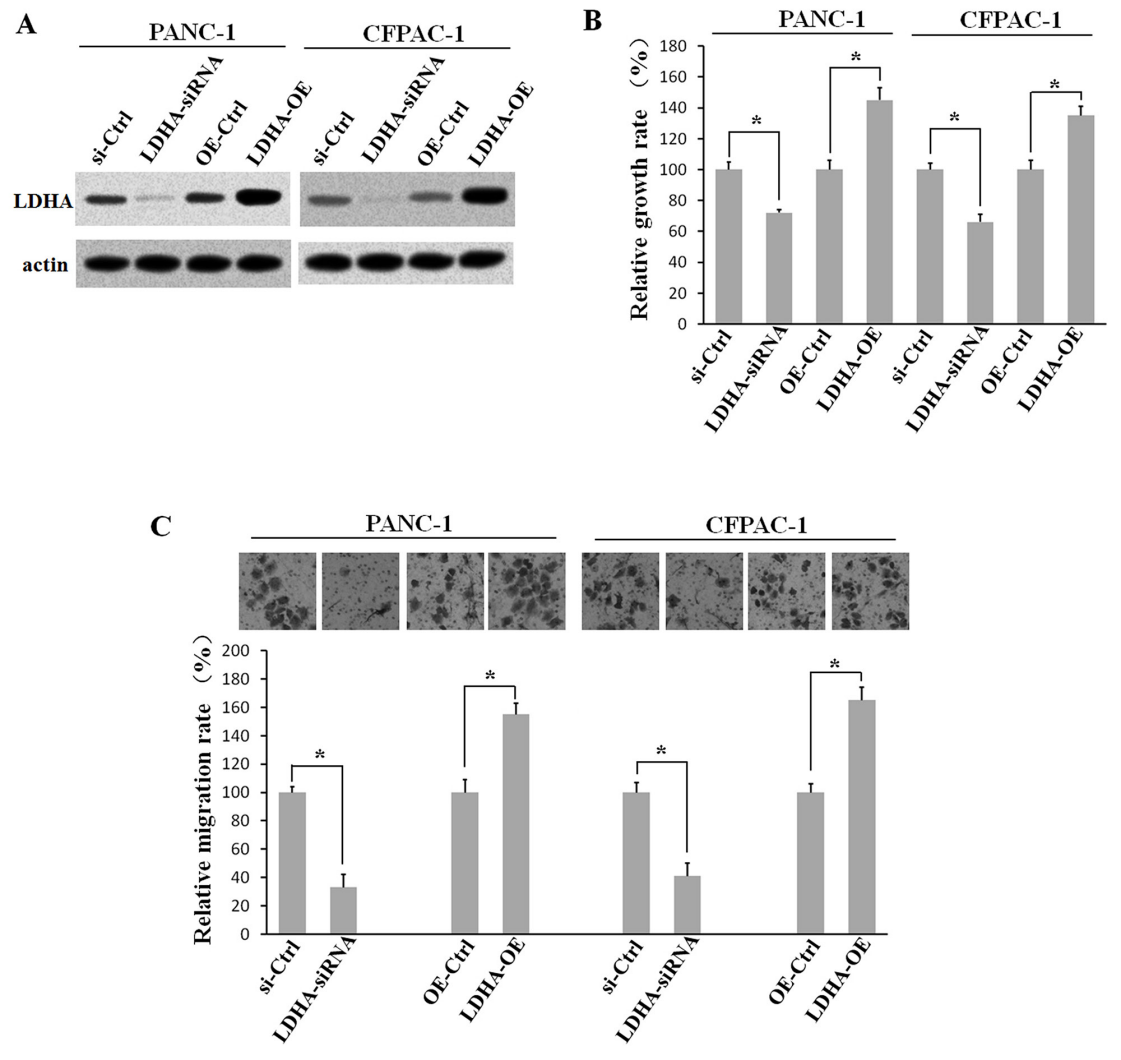
LDHA promotes PC cell growth and migration PANC-1 and CFPAC-1 cells were transfected with pcDNA3.1-HA-LDHA or siRNA. **A**. Western blot analysis showing the expression of LDHA. **B**. CCK8 assays were performed to inspect the growth of pancreatic cells after alteration in LDHA expression. **C**. Transwell migration assays were applied to detect the cell migration ability. *: p < 0.01 (Student's t-test).

To examine the effect of LDHA on PC cells, PANC-1 and CFPAC-1 cells were transfected with pcDNA3.1-HA-LDHA or siRNA. Western blot analysis was applied to determine the expression of LDHA (Figure 7A). Then, CCK8 assay was performed to observe PC cell growth after alteration of the LDHA expression. The results showed that LDHA over-expression promoted PC cell growth, while knocking down the expression of LDHA inhibited cell growth markedly (Figure 7B). Further Transwell migration assay showed that silencing the expression of LDHA inhibited PC cell growth, and LDHA over-expression promoted cells migration (Figure 7C). These result showed that LDHA played an oncogenic role in PC.

## DISCUSSION

The morbidity of PC is increasing steadily because of the high malignant nature of PC and the relatively low curative effect [19]. The key to successful targeted molecular therapies against this lethal malignancy is the identification of critical and active oncogenes in oncogenic networks. Tumor energy metabolism always under the hypoxic condition, and the way of energy generation is crucial for tumor development, invasion and metastasis. Hypoxia, HIFs and LD all contribute to this energy metabolism, to explore the relationship in these factors and study the underline mechanism is very necessary for fighting pancreatic cancer.

Intra-tumor hypoxia is a characteristic micro-environmental factor in malignant tumors that drives tumor progression [20]. In a hypoxic condition, most cancer cells predominantly produce energy at a high rate of glycolysis [21]. LDHA catalyzes the final step of glycolysis in which pyruvate converted to lactate, with concomitant inter-conversion of NADH and NAD+ [22]. It has long been known that LDHA levels in many human cancers including breast cancer, melanoma, renal cell carcinoma and gastric cancer are significantly higher than those in normal tissues [23–25]. Previous studies [26, 27] demonstrated that LDHA promoted the development, invasion and metastasis of malignancies [26, 27]. Knockdown of LDHA in tumor cells by shRNAs led to an increase in mitochondrial respiration, a decrease in cell proliferation under hypoxia, and suppression of tumorigenicity [28]. LDHA is regarded as a potential prognostic biomarker, and over-expression of LDHA is closely correlated with intrahepatic metastasis, early recurrence and worse prognosis in colon cancer [29]. Therefore, manipulation of LDHA expression may have impact on cancer cell proliferation, viability or invasiveness.

In the present study, we analyzed 244 PC specimens and adjacent normal tissues and found that the expression of LDHA was elevated in PC significantly. In addition, the expression of LDHA was closely associated with the development and progression of PC. Then, we treated PANC-1 and CFPAC-1 cells under the hypoxic condition (0.1% O2) and found that LDHA was over-expressed at both mRNA and protein levels, indicating that hypoxia induced the expression of LDHA in human PC cell lines.

HIF is a transcriptional regulator that mediates cellular and systemic adaptive responses to maintain oxygen homeostasis in all metazoan species. HIF is composed of an oxygen-regulated α-subunit and a constitutively expressed β-subunit. HIF-α and HIF-β integrate dimer bind to specific DNA sequences within the promoter of target genes called HREs, which are composed of 5′-RCGTG-3′ and recruit co-activators [30, 31]. In the normoxia condition, HIF-α and HIF-β undergo posttranslational modification by oxygen-dependent prolyl and asparaginyl hydroxylases that decrease HIF-αprotein stability and activity, respectively [32, 33]. When oxygen levels are low, oxygen-dependent hydroxylases are inactivated and HIF-a remains in a stable state. It was found in our study that both HIF-1 and HIF-2 were over-expressed when PANC-1 and CFPAC-1 cells were subjected to hypoxia. It was reported [34–36] that increased levels of HIF-1 or HIF-2 were correlated with adverse prognosis in breast, cervical, endometrial, colorectal, NSCLC, ovarian, rectal, pancreatic and prostate cancers. We also found that both HIF-1α and HIF-2α were increased in PC specimens. Although how HIF-1 or HIF-2 contributed to the adverse prognosis remains unclear, the transcriptional effect on glycolytic enzymes may be the potential mechanism.

HIF-1 and HIF-2 have their unique targets contributing to the characteristics of these two proteins. It was reported [41–43] that HIF-1 preferentially induced genes in the glycolytic pathway and HIF-2 was involved in the regulation of genes important for tumor growth, cell cycle progression and maintaining stem cell pluripotency [41, 42, 43]. Meanwhile, they also share some similar properties through transcription of a group of common target genes. Erythropoietin (EPO) and vascular endothelial growth factor (VEGF) are known to share target genes of HIF-1 and HIF-2 [44].

It was found in our study that there was a strong association between HIF-1α and HIF-2α over-expression and LDHA over-expression in human PC specimens (n = 244, p < 0.0001). Ectopic HIF-1α and HIF-2α expression transcriptionally induced the expression of LDHA. Knockdown of endogenous HIF-1α or HIF-2α alone largely decreased the hypoxia-induced LDHA level, and knockdown of both HIF-1α and HIF-2α reversed this effect, implying that LDHA is a common target gene of both HIF-1 and HIF-2. Currently, there are few studies addressing the relationship between HIF-2 and LDHA. To the best of our knowledge, this is the first study to discover that HIF-2 regulates LDHA in PC. The LDHA promoter contains 5 putative HER sites (HER-A, HER-B, HER-C, HER-D and HER-E). CHIP and Luciferase Reporter Assay showed that binding of both HIF-1α and HIF-2α to HER-D activated LDHA transcription regardless of the hypoxic condition or HIF-1α and HIF-2α over-expression. Mutant HER-D reversed the effect of hypoxia on LDHA over-expression. To the best of our knowledge, this is the first study to identify the accurate binding site in LDHA for both HIF-1α and HIF-2α, which may contribute to a better understanding about the mechanism underlying energy metabolism in PC and other cancers with “Warburg effect” as well. Furthermore, forced expression of LDHA promoted the growth and migration of PC cells, while knocking down the expression of LDHA inhibited cell growth and migration markedly, suggesting that LDHA plays an oncogenic role in PC cells.

In summary, our study not only provided explicit evidence that both HIF-1 and HIF-2 mediate hypoxia-induced LDHA expression in human PC but determined the specific binding site of HIF-1 and HIF-2. In addition, LDHA was found to play an oncogenic role in promoting PC growth and migration. The study may provide a wider perspective for PC research and treatment.

## MATERIALS AND METHODS

### Patients and tissue specimens

A total of 244 PC specimens were obtained from patients who underwent pancreatectomy between January 2010 and October 2015 at the Department of Surgery in Changzheng Hospital of the Second Millitary Medical University (Shanghai, China). None of the patients in our study received neoadjuvant chemotherapy. Patient data including age, gender, tumor size and TNM classification are summarized in Table 1. The histological differentiation and histological types of these specimens were assessed by experienced pancreatic pathologists.

### Immunohistochemical (IHC) and immunofluorescence colony (IFC) staining assays

Tumor tissues or other samples were fixed with 4% paraformaldehyde, dehydrated through a graded series of ethanol, paraffin embedded, sliced into 5-μm sections, IHC stained, and scored for LDHA according to the manual of the Histostain-Plus (DAB) kit (Mingrui Biotech) by two investigators independently who were blind to the histologic grade of PC specimens. The specimen was scored as negative (0), weak (1+), moderate (2+), or strong (3+).

IFC staining was performed with primary and secondary antibodies diluted in 10% BSA, and the nucleus was stained by DAPI (4′,6-diamidino-2-phenylindole, Sigma). All fluorescent secondary antibodies were used at a dilution of 1:200 for 30 min (invitrogen). Quantification of IFC was performed with NIH Image J.

### Cell culture and treatment

Human PC cell lines PANC-1 and CFPAC-1 were obtained from the American Type Culture Collection (ATCC). Cells were cultured in DMEM supplemented with 10% fetal bovine serum (FBS, Sigma). For hypoxic treatment, cells at 50-60% confluence were incubated in a hypoxia chamber (0.1% O2, Billups-Rothenberg). HIF-1α expression plasmids (pcDNA3.1-HA-HIF-1α), HIF-2α expression plasmids (pcDNA3.1-HA-HIF-2α) and LDHA expression plasmids (pcDNA3.1-HA-LDHA) were obtained from Addgene. For siRNA knockdown, three different siRNA oligos against HIF-1α, HIF-2α and LDHA were purchased from IDT. siRNA targeting HIF-1α: siRNA-1: 5-CAG ACU UUA UGU UCA UAG UUC UUC CUC-3; siRNA-2: 5-CUU CCA CAA CUA CAU AGG GUA UUG UUU-3. siRNA targeting HIF-2α: siRNA-1: 5-AUA CAG UUA UAA UGU UGU CAG UAG GAA-3; siRNA-2: 5-UCA UUG AAA UCC GUC UGG GUA CUG CAU-3. siRNA targeting LDHA:5′-TTG TTG ATG TCA TCG AAG-3′ and 5′-GGG TCC TTG GGG AAC ATG-3′. Expression plasmids and siRNA oligos were transfected into cells using X-tremeGENE HP (Roche).

### RT-PCR

Total RNA was isolated by using RNeasy Kit (Qiagen) following the manufacturer's instruction. RNA was reverse transcribed into cDNA by using the Taqman Reverse Transcription Reagents kit (Applied Biosystems) with random hexamers. Human LDHA, VEGF, HIF-1α, HIF-2α and actin mRNA levels were determined in Stepone Plus real-time PCR System (Applied Biosystems). All primers were purchased from Applied Biosystems. Real-time PCR was done in triplicate with TaqMan PCR mixture (Applied Biosystems). The expression of genes was normalized to the actin gene.

### Western-blot assays

Standard Western-blot assays were used to analyze the levels of protein. Antibodies against HIF-1α (sc-13515, Santa Cruz Biotechnology, 1:500), anti-HIF-2α (sc-13596, Santa Cruz Biotechnology, 1:500), anti-LDHA (ab84716, Abcam, 1:500 dilution) and anti–β-actin (A5441, Sigma) antibodies were used in this study.

### Construction of reporter plasmids

The fragments containing the potential HRE sites (5-G/ACGTG-3) identified from the LDHA promoter (A-E:-1292bp∼553bp; B-E: -622bp∼553bp; C-E: -116bp∼553bp; D-E: 2bp∼553bp; E: 131bp∼553bp) and VEGF promoter (from-1080bp to -874bp upstream of transcription initiation site) were cloned into pGL3-reporter Luciferase Reporter Vector (Promega) at KpnI-XhoI restriction sites using In-Fusion cloning Kit (TAKARA). All constructs were confirmed by DNA sequencing.

### Luciferase activity assay

To study whether hypoxia transactivated the pGL3-reporter plasmids described above, cells were transiently transfected with the pGL3-reporter plasmids containing one copy of each potential HRE site (or mutate HRE, Figure 3D) together with pRL-null (Promega) as an internal control to normalize transfection by using X-treme GENE HP (Roche). Cells were then subjected to hypoxia treatment for 48 h. To study the transactivation activity of HIF-1/2α on pGL3-reporter plasmids, cells were co-transfected with pGL3-reporter plasmids together with HIF-1/2α expression plasmids. The luciferase activity was measured by using the Dual Luciferase assay kit (Promega) and normalized with the internal standard.

### ChIP assays

ChIP assays were performed by using a ChIP assay kit (Millipore) in accordance with the instructions of the manufacturer. In brief, cells cultured under the hypoxic or normaxic conditions or transfected with HIF-2α expression plasmids were subjected to ChIP assays with anti-HIF-1α or anti-HIF-2α antibodies. Normal IgG was used as a control for nonspecific binding of genomic DNA. DNA fragments pulled down by antibodies were recovered and subjected to real-time PCR and conventional PCR by using the PCR primer sets described above with the omission of restriction enzyme recognition sequence.

### Lactate production and glucose utilization assay

Pancreatic cancer cells were transfected with plasmids and siRNAs, and 1 × 106 cells were prepared for Lactate production assay with the Lactate Assay Kit (Sigma, Louis, MO, US) according to the manufacturer's protocol. For glucose utilization assay, tumor cells were transfected with plasmids and siRNAs, and the cultures were incubated for 24 h. The media were replaced with phenol-red free RPMI with 1% FBS or phenol-red free RPMI with 1% FBS and 20 mmol/L oxamate to continue the culture for 3 days. Glucose concentrations in media were measured by a colorimetric glucose assay kit (Biovision, US) and normalized with cell number [45].

### CCK8 assay

Cells that transfected with OE-LDHA plasmids or siRNA for 24 h were digested and counted. Then, cells were seeded in 96-well plates and cultured for 48 h and assessed using the Cell Counting Kit 8 (biotool, USA). Results were measured by absorbance at 450 nm using an ELx800 microplate reader (BioTek Instruments Inc., USA).

### Transwell migration assay

An 8 μm pore size transwell chamber without matrigel (3422, Corning, USA) was used for transwell migration assay. Cells transfected with OE-LDHA plasmids or siRNA for 24 h were digested and counted. A total of 1×105 cells in 100μL medium supplemented with no FBS were plated in the upper chamber and 500μL medium supplemented with 10% FBS was covered on the bottom chamber as chemoattractant. After 24-h incubation in a humidified incubator, non-migratory cells on the upper membrane surface were carefully removed, and those on the bottom surface were fixed with 4% polyoxymethylene and stained with 0.1% crystal violet for 15 min. Cells were counted by photographing 5 random fields under a microscope at 400× magnification.

### Statistical analysis

SPSS 20.0 statistical software (SPSS Inc., Chicago, IL) was used for statistical analysis. The data were expressed as mean ± SD. The correlation between HIF-1α, HIF-2α and LDHA expression levels was analyzed by Fisher exact test. All other p values were obtained through two-tailed Student's t-test. Values of p < 0.05 were considered to be statistically significant.
